# Visible light communication with efficient far-red/near-infrared polymer light-emitting diodes

**DOI:** 10.1038/s41377-020-0314-z

**Published:** 2020-04-26

**Authors:** Alessandro Minotto, Paul A. Haigh, Łukasz G. Łukasiewicz, Eugenio Lunedei, Daniel T. Gryko, Izzat Darwazeh, Franco Cacialli

**Affiliations:** 1 Department of Physics and Astronomy and London Centre for Nanotechnology, University College London, London, WC1E 6BT UK; 2 School of Engineering, Newcastle University, Newcastle-upon-Tyne, NE1 7RU UK; 3 Communications and Information Systems, University College London, London, WC1E 6BT UK; 4 Institute of Organic Chemistry, Polish Academy of Sciences, 01-224 Warsaw, Poland; 5 ISMN-CNR, Institute for the Study of Nanostructured Materials, 40129 Bologna, Italy

**Keywords:** Organic LEDs, Fibre optics and optical communications

## Abstract

Visible light communication (VLC) is a wireless technology that relies on optical intensity modulation and is potentially a game changer for internet-of-things (IoT) connectivity. However, VLC is hindered by the low penetration depth of visible light in non-transparent media. One solution is to extend operation into the “nearly (in)visible” near-infrared (NIR, 700–1000 nm) region, thus also enabling VLC in photonic bio-applications, considering the biological tissue NIR semitransparency, while conveniently retaining vestigial red emission to help check the link operativity by simple eye inspection. Here, we report new far-red/NIR organic light-emitting diodes (OLEDs) with a 650–800 nm emission range and external quantum efficiencies among the highest reported in this spectral range (>2.7%, with maximum radiance and luminance of 3.5 mW/cm^2^ and 260 cd/m^2^, respectively). With these OLEDs, we then demonstrate a “*real-time*” VLC setup achieving a data rate of 2.2 Mb/s, which satisfies the requirements for IoT and biosensing applications. These are the highest rates ever reported for an online unequalised VLC link based on solution-processed OLEDs.

## Long-wavelength OLEDs: visible light communication

Highly efficient organic light-emitting diodes (OLEDs) that operate in the red and near-infrared region bring new opportunities for applications involving visible light communication (VLC). Alessandro Minotto and coworkers from the UK, Poland and Italy have designed and fabricated heavy-metal-free OLEDs featuring a polymeric light-generating active region composed of a fluorescent 𝜋-expanded diketopyrrolopyrrole dye called eDPP blended in a host charge-transport matrix called F8BT. The devices emit light at 650–800 nm with an external quantum efficiency of around 2.7% and a radiance of 3.5 mW/cm^2^. When employed in a data communications link, error-free data rates of ~2.2 Mb/s were achieved making them suitable for a variety of internet-of-things (IoT) applications such as data-enabled biosensing implants where shorter-wavelength visible light cannot be used due to the issue of strong absorption.

## Introduction

In recent years, the increasing demand for faster data transmission speeds has shifted the attention of researchers from bandwidth-limited radio technologies to optical wireless communication systems^1^, which offer “practically” unlimited bandwidth (>400 THz) by exploiting the ultraviolet to infrared region of the electromagnetic spectrum. Among these systems, VLC systems^2^ are appealing because of the possibility of leveraging the ubiquity of the light-emitting devices already used in countless commercial applications, ranging from lighting systems to mobile phones and TV displays.

In the fastest VLC links reported thus far, the optical transmitters consist of light-emitting diodes (LEDs)^3^ and laser diodes (LDs)^4–6^ featuring an inorganic semiconductor (typically gallium nitride) as the emissive medium, which affords high optical output power and broad bandwidth. These properties make inorganic LEDs^1,2^ and LDs^7,8^ suitable for integration into dual-purpose luminaires capable of simultaneously providing white lighting and data transmission. However, the use of organic light-emitting diodes (OLEDs) represents a valid alternative, which is gaining considerable attention for VLC^9–14^. The interest in OLEDs for communication is driven not only by their widespread use in display technologies but also by the very same advantages that have been driving the success of organic electronics. Above all, organic semiconductors (OSs) can be cheaply deposited over large areas, either via thermal evaporation or from solution via spin^15^, blade^16^, inkjet^17^, or spray coating^18^. Large area OS-based luminaires are already entering the market^19^ and are expected to be the next mass application of OSs after active-matrix OLED (AMOLED) displays.

VLC links integrating OLEDs as optical transmitters have already been reported by our groups and others^9–14^, with data rates exceeding 10 Mb/s due to the leveraging of both equalisation algorithms and wavelength division multiplexing. Undoubtedly, such data rates are not as high as those afforded by “inorganic” VLC systems (up to 35 Gb/s)^20^ based on multiple-quantum-well (MQW) LEDs and LDs, which are mainly limited by the intrinsic exciton decay lifetime^21–24^. This lifetime can be decreased to the sub-nanosecond range by engineering the MQW active layer volume, improving heat sinking, heavy doping, or using non-polar substrates^21–24^. As discussed in previous reports^9–14^, OLEDs’ lower modulation bandwidths arise from their RC time constant, which strongly depends on charge mobilities of ~10^−6^–10^−^^2^ cm^2^/V s in OSs (at least three orders of magnitude lower than that in GaN) and the size of the photoactive area^13^. However, these mobilities are still suitable for a wide range of prospective connections, such as those expected for the implementation of the internet-of-things (IoT), which only require data rates of a few Mb/s (or less). This circumstance thus shifts the “figure of merit” emphasis from data rates to specifications such as cheap fabrication over large areas, toxicity, recyclability and/or sustainability, production volume, and flexibility, for which organics are better suited.

When considering the downlink and uplink capacities, more robust transmitters (i.e., inorganic LEDs) are generally selected for the downlink since they currently offer higher power output than OLEDs, although in the future OLEDs may offer similar power levels and be an alternative. However, we envisage OLEDs integrated into low-cost, high-performance display technologies as the uplink in VLC-based 5G access links, whose standards currently define the downlink-to-uplink speed ratio as ranging from 4:1 to 8:1, depending on the traffic volume^25^. Such a reduced throughput requirement, coupled with the SNRs and data rates demonstrated in our previous work^12^, proves that data rates sufficiently high to satisfy uplink requirements can be supported.

Extending the spectral range into the near-infrared (NIR, 700–1000 nm) window not only expands the bandwidth of VLC links but also paves the way for their integration into many applications exploiting NIR radiation. NIR-emitting devices are used in several different fields (please refer to ref. ^26^ for a more extensive review), including security, biodetection and photodynamic/photothermal therapy, with the latter two benefitting from the semitransparency of biological tissue in this spectral window. In the case of wearable or implanted biosensors, NIR photons can be used to both monitor human vital signs and wirelessly communicate with other devices.

Achieving high fluorescence from OSs in the NIR is particularly hard because two major challenges must be overcome^26^. First, to obtain NIR emission, the energy gap of the chromophore must be reduced, thereby increasing the probability of non-radiative exciton decay (the so-called “energy-gap law”)^27^. Second, to reduce the gap, chromophores are designed to exhibit higher planarity and extended conjugation length with respect to visible emitters, ultimately favouring the undesired formation of poorly emissive molecular aggregates^28^.

Despite these hurdles, NIR OLEDs present some advantages compared with inorganic NIR emitters, offering both mechanical conformability and design freedom afforded by low-cost wet deposition methods. Crucially, organics also offer material design flexibility, preferentially developed in the direction of heavy-metal-free materials because these materials are both more environmentally sustainable and biocompatible, and thus preferred for biomedical and/or food industry applications in which toxicity is an issue. Narrow-gap hybrid organic–inorganic materials and organometallic phosphorescent complexes^26,29,30^, even those characterised by external quantum efficiencies (EQEs) exceeding 10%, are thus less appealing. Interestingly, EQEs in the 1–10% range have also been reported for devices incorporating heavy-metal-free (purely organic) NIR fluorescent dyes^26,31–33^. However, the most efficient of these approaches leverage, to a significant extent, triplet-to-singlet conversion (so-called triplet–triplet annihilation (TTA) or reverse inter-system crossing (rISC)) to obtain high efficiency, with a concomitant intrinsic limitation of the bandwidth even when the problems of the RC time constant can be overcome by reducing the device area^13^. The maximum EQEs for NIR-emitting devices in which triplet leveraging plays a minor or no role have been limited to 1.2%^32,33^.

**Fig. 1:**
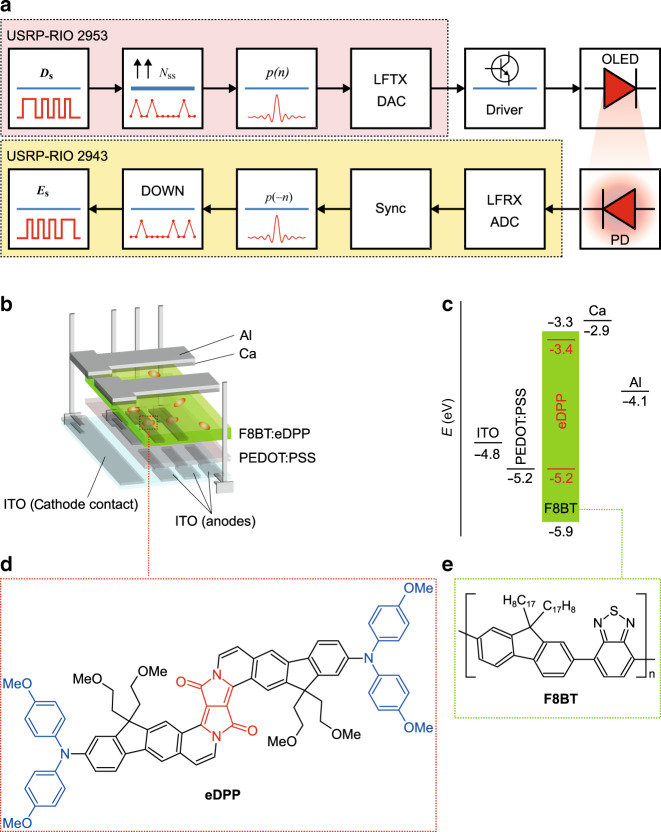
Red/NIR-emitting OLEDs for VLC. **a** Simplified block diagram of the real-time experimental VLC setup including pulse shaping *p*(*n*) and matched filtering *p*(−*n*) (where *n* is the current discrete time sample) using a square-root raised cosine, as defined in ref. ^10^. Acronyms are defined as follows: USRP-RIO, Universal Software Radio Peripheral–(with) reconfigurable input/output, emulating data traffic in the on–off keying transmission mode at the transmitter (2953) and the receiver (2943); *D*_s_ data source, *N*_ss_ number of samples per symbol, LFTX/RX low-frequency transmitter/receiver, DAC digital-to-analogue converter, PD ThorLabs PDA36A-2 silicon detector, ADC analogue-to-digital converter, Sync synchronisation, DOWN down-sampling, *E*_s_ estimated data source, OLED organic light-emitting diode. **b** ITO/PEDOT:PSS/F8BT:eDPP/Ca/Al OLED architecture, including an indium tin oxide (ITO) patterned substrate (anode), a poly(3,4-ethylenedioxythiophene) doped with poly(styrene sulfonate) (PEDOT:PSS) hole-transport layer, an F8BT:eDPP red/NIR-emitting layer, calcium/aluminium (Ca/Al, cathode) and electrode connection legs. **c** Band diagram for the materials employed in the OLEDs^33^. **d** Molecular structure of the far-red/NIR-emitting π-extended DPP (eDPP). **e** Molecular structure of the F8BT host matrix

In this work, we report full optoelectronic characterisation of a new class of far-red/NIR OLEDs (Fig. 1) incorporating a fluorescent π-expanded diketopyrrolopyrrole dye (eDPP) blended in a poly(9,9-dioctylfluorene-*alt*-benzothiadiazole) (F8BT) charge-transport matrix. F8BT:eDPP OLEDs show electroluminescence (EL) peaking at 670 nm, with ~50% of photons falling in the NIR spectral range (λ > 700 nm), radiances >3 mW/cm^2^, and EQEs reaching 2.72%. We capitalised on these results, unprecedented for an OLED incorporating a metal-free fluorescent dye emitting in this spectral range, by integrating such devices into a real-time VLC setup, whose simplified block diagram is shown in Fig. 1a, reaching error-free transmission speeds in excess of 1 Mb/s.

To the best of our knowledge, such data rates are the highest ever reported for an online VLC link based on organic LEDs without the use of computationally complex equalisers. For practical real-time systems, increasing the transmission speed and demonstrating high transmission speeds without equalisation are attractive prospects for a broad range of printable/portable/wearable/implantable NIR-based applications, such as in photonic biosensing^34^ or enabling low-power IoT connectivity^35^, among others (e.g., but not limited to, food packaging, with the tantalising prospect of integrating both sensing and IoT). Due to their high degree of complexity, high-performance equalisers require sophisticated digital signal processing circuitry, which will negatively impact the battery life and increase the required physical surface area at the receiver, which is not desirable for such applications. Similarly, “real-time” is more appealing than “offline” signal processing, as the latter does not take into account numerous practical implementation issues, such as finite resources, fixed point processing and restricted sampling frequencies, and is generally used at the proof-of-concept stage. Real-time signal processing is a significant challenge to effectively realise and represents the true performance of the link.

## Results

The eDPP red/NIR emitter was synthesised following the procedure reported by Grzybowski et al.^36^, starting from a suitably substituted diketopyrrolopyrrole (DPP) (Fig. 1d). DPP derivatives are versatile dyes exhibiting a favourable combination of optoelectronic properties^37–39^ that have been extensively investigated and exploited in a very broad range of applications, including organic transistors^40^, solar cells^41^ and OLEDs^42^. Interestingly, the optical properties of DPP can be drastically modified by leveraging its highly electron-poor nature and the so-called “push–pull” approach to obtain red-to-NIR-emitting derivatives that combine both enhanced non-linear optical properties and high photoluminescence (PL) quantum efficiencies^43^.

In the case of eDPP, both its ten-ring-fused rigid structure and the presence of fluorene and diarylamino electron-donor units on either side of the electron-accepting DPP moiety afford extended electron delocalisation and a small energy gap (~1.8 eV, as measured via cyclic voltammetry, see Methods). Furthermore, the alkyl chains of the fluorene units confer good solubility to eDPP in comparison to, for example, similar highly planar DPP ring-fused derivatives^36^, thus crucially facilitating its incorporation into a polymeric matrix. Furthermore, the presence of two fluorene units in the backbone makes eDPP suitable for blending into polyfluorenic matrices to mitigate aggregation quenching and facilitate charge transport.

Among the plethora of fluorene-based polymers available on the market, we selected F8BT as a matrix because the relatively deep-lying lowest unoccupied and highest occupied molecular orbital energy levels (LUMO and HOMO, respectively, essentially the equivalent of the conduction and valence bands for inorganic semiconductors) enable efficient electron injection (into the LUMO) and, concurrently, the favourable formation of a “so-called” type-I heterojunction^32,33^ with eDPP (Fig. 1c). Such a type-I heterojunction also proved effective with other dyes^32,33,44^ by favouring exciton localisation (in the emitter) over undesired exciton dissociation. Contrary to the case of eDPP, in F8BT, TTA^45^ and rISC^46^ contribute to the formation of a minority of singlet excitons (with subsequent transfer to eDPP) over a timescale of ~1 μs^47–49^, but this does not compromise application to VLC, as most of the EL decay is prompt and only 10% of the initial intensity decays over timescales longer than a few hundreds of nanoseconds (vide infra).

### Optical characterisation

**Fig. 2:**
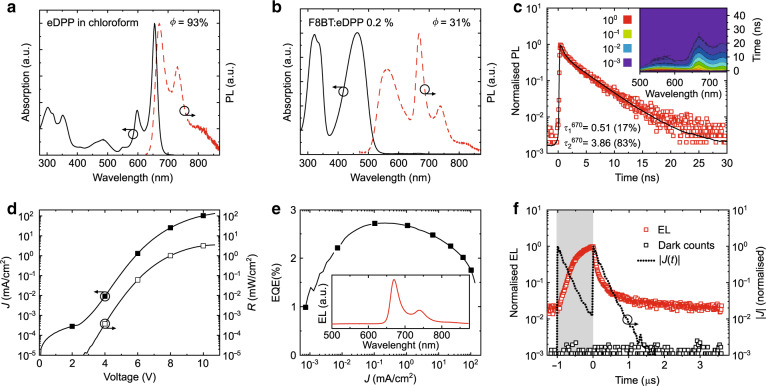
Photoluminescence and electroluminescence properties of eDPP and of the F8BT:eDPP blend with 0.2 wt% eDPP loading. **a** Normalised absorption and photoluminescence (PL) spectra of eDPP in chloroform (at an ~10^−^^6^ M concentration). **b** Normalised absorption and PL of thin films (~125 nm) of the F8BT:eDPP 0.2 wt% blend (on Spectrosil). The PL spectra in **a** and **b** were obtained by exciting the samples at 375 and 445 nm, respectively. The PL quantum yield (*ϕ*) values are reported at the top right of each plot. **c** Semi-log plot of the transient PL at 670 nm (excitation at 445 nm with a ps pulsed laser) and time-resolved PL spectra (inset). The black line is the fit to the experimental data (red open squares) obtained via iterative reconvolution of a biexponential equation with the instrument response function. The time constants (and the corresponding percentage weights) of each exponential component extracted from the fit are shown in the bottom left-hand corner. All steady-state and transient spectra in **a**–**c** were measured in air at room temperature. **d**, **e** Current density and radiance vs. voltage (*JVR*) characteristics (**d**), and external quantum efficiency (EQE) vs. current density (*J*) plot (**e**). The electroluminescence (EL) spectrum (inset in **d**) was measured at 5.2 V (i.e., where the EQE is maximum, EQE_MAX_ = 2.72%) for a device with an eDPP:F8BT layer thickness of ~125 nm, a PEDOT:PSS thickness of ~40 nm, and a Ca(30 nm)–Al(200 nm) cathode. **f** Semi-log plot of the transient EL at 670 nm and background signal (red and black squares, respectively, both normalised to the EL maximum) measured by exciting the OLEDs with 4.8 V rectangular voltage pulses (200 kHz repetition rate and 1 μs pulse width, shown as the grey shading in **f**). The dotted line in **f** represents the absolute value of the normalised transient current (|*J*(*t*)|) flowing across the circuit during the OLED pulsed operation

We studied the absorption and luminescence properties of eDPP both in solution and in the solid state. The main results are summarised in Fig. 2 and in the Supplementary Information section (Supplementary Fig. S1, Supplementary Table S1).

As shown in Fig. 2a, the absorption of eDPP in the UV–visible spectral range is dominated by a well-resolved vibronic manifold in the 530–700 nm region, corresponding to the S_0_ → S_1_ electronic transition, and by a secondary band at higher energy (peak at 300 nm) due to S_0_ → S_*n*_ transitions, which is typical for π-expanded diketopyrrolopyrroles^36,37^. The maximum of the S_0_ → S_1_ vibronic progression at 655 nm is due to the narrow (61 meV full-width at half-maximum) purely electronic 0–0 transition. Note also the small Stokes shift (only 42 meV) and the clear symmetry of the vibronic structure between absorption and fluorescence (peaking at 670 nm). We attribute such a small shift to the rigidity of the ten-fused-ring structure of eDPP, which suppresses structural relaxation in the excited state with respect to the “quasi-planar”^36^ ground-state conformation. Ultimately, both the extended π-conjugation and the excited-state planarity favour high oscillator strength (*f* = 0.83)^36^ and fluorescence quantum efficiency (*ϕ* = 93 ± 4% in chloroform), similar to other conjugated systems, such as rigid rod-like ladder-type poly(*para*-phenylene)^50^ and porphyrin oligomers^44,51^.

Interestingly, the 0–1 and 0–2 transitions fall well within the NIR range, peaking at 730 and 800 nm, respectively, yielding an overall 58% of photons being emitted in the NIR. Such a high fraction of NIR emission, combined with the almost 100% fluorescence efficiency, makes eDPP one of the most efficient NIR dyes emitting above 700 nm^52^ reported thus far.

Effective exploitation of eDPP in OLEDs requires further optimisation of the blend concentration to reach the best compromise between the opposite needs of suppressing aggregation and concentration quenching (typically by reducing the dopant/emitter concentration in the host to approximately <10 wt%) and of ensuring efficient energy transfer from the host to quench its luminescence and afford colour purity. To find the best trade-off, we thus investigated thin films with four different eDPP loadings, namely, 0.2, 1, 2.5 and 10 wt%, and report full details of their absorption and PL in the Supplementary information (Section 1).

Surprisingly, while we found a remarkable 45% maximum PL efficiency (*ϕ*) for the 2.5 wt% blend, with only 7% residual green emission from F8BT in the PL (Table S1), the 0.2 wt% blend thin films displayed a lower PL efficiency (*ϕ* = 31 ± 2%) but outperformed all the other films in terms of the EL efficiency and optoelectronic signalling performance in OLEDs (while maintaining <1% F8BT green emission in the EL). This is a slightly counterintuitive result, especially compared with the majority of previously reported concentration studies, in which the blends with the lowest NIR dye loading typically exhibited the highest PL (and EL) efficiency due to the suppressed aggregation quenching^26^. The apparently unusual result we report here can be accounted for by noting that when the PL efficiency of the guest in solution (in our case ~93%) is significantly higher than that of the host (~22% for F8BT thin films), the PL efficiency is not necessarily a monotonic decreasing function of the (increasing) concentration. This is because for decreasing guest concentration, a greater fraction of emission occurs from the lower efficiency host as a result of the less efficient energy transfer. Similarly, owing to the interplay between (energy selective) charge transport and luminescence during EL operation, both the emission spectra and optimum concentrations need not be the same for EL and PL (vide infra).

In fact, by examining the PL characteristics summarised in Table S1 in more detail, 0.2 wt% turns out to be the optimal eDPP concentration for minimising aggregation quenching and affording good spectral purity. Namely, when taking into account the large (~50%) contribution of F8BT to the overall PL (consistent with an energy transfer efficiency of ~17% at 0.2 wt% loading, as measured via transient PL experiments) and *ϕ* = 22% for F8BT (as a neat undoped film, for the particular batch used here), one would expect an overall decrease in the efficiency of the blend down to ~34%. Strikingly, while such a maximum theoretical efficiency (*ϕ*^th^ in Table S1) of the blend essentially matches the experimentally obtained *ϕ* = 31 ± 2% for the 0.2 wt% blend, *ϕ* is markedly lower than *ϕ*^th^ at higher eDPP concentrations, proving that detrimental intermolecular interactions are suppressed only at eDPP loadings as low as 0.2 wt%. Furthermore, this PL characterisation result not only translates into the 0.2 wt% eDPP OLEDs being the most efficient ones (vide infra) but also proves that a further reduction of the dopant concentration would only lead to a lower energy transfer rate and hence higher residual emission from the F8BT host (i.e., lower colour purity), with a negligible effect on the eDPP radiative efficiency.

For this reason, in the following, we focus on 0.2 wt% blends (and devices) and use optical spectroscopy first of all to gain insight into their photophysics when switching from solutions to films.

First, it is interesting that although the blend absorption profile (Fig. 2b) is dominated by F8BT’s bands (460 and 325 nm), eDPP’s absorption and emission features are still clearly visible on the appropriate scale (e.g., see the zoomed-in image in Supplementary Fig. S1c), with an essentially unaltered vibronic progression in films vs. solutions (Fig. 2b, Supplementary Fig. S1). This confirms that the influence of intermolecular interactions (either due to other eDPP molecules or F8BT chains) on the optical properties of eDPP is essentially negligible for the 0.2 wt% blend.

Investigation of the exciton dynamics via time-resolved spectroscopy powerfully corroborates this conclusion on the basis of a dominant (82% weight) component with a 3.86 ns exciton lifetime (Fig. 2c, measured at the eDPP PL maximum = 670 nm), essentially comparable to the one found in solution (3.68 ns; Supplementary Fig. S1, Supplementary Table S1). We attribute the slight increase (~0.2 ns, i.e., <5%) in lifetime to residual intermolecular interactions and to a subsequent reduction of the radiative rate. At such a low concentration, we can also detect an additional and faster component (0.51 ns, relative weight of 18%, observable in Fig. 2c), which we assign to the quenched F8BT emission. This component is no longer visible in higher concentration blends (Supplementary Fig. S1, Supplementary Table S1) due to the more efficient (and faster) quenching via resonant energy transfer from F8BT to eDPP.

Importantly, we reiterate that the relatively large F8BT contribution to the PL of the 0.2 wt% blend does not compromise the colour purity of the EL emission owing to the energy selective nature of charge transport combined with the type-I heterojunction energetics of our blends. These factors ensure that excitons are formed preferentially on the guest and ultimately that the EL from F8BT remains at <1% of the overall emission, as detailed below.

### OLEDs DC characterisation

OLEDs incorporating 0.2 wt% eDPP in the active layer turn on at a *V*_on_ of ~2.8 V or slightly less, lower than the value expected for doped F8BT OLEDs (which usually display higher *V*_on_ than neat F8BT devices)^33,44,47–49^, with the maximum radiance reaching 3.5 mW/cm^2^ at 10 V (the results for the other blends are given in the Supplementary Information, Section 2, Supplementary Figs. S2 and S3).

The EL spectrum falls in the red/NIR spectral region and peaks at 670 nm (inset of Fig. 2e). As anticipated, F8BT emission is nearly absent here (<1%), thus enabling virtually pure red/NIR emission. This occurs because during transport charges preferentially localise on guest (eDPP) molecules (owing to the type-I heterojunction energetics^32,33^), which in turn act as traps for the oppositely charged carriers.

We measured a maximum EL EQE of 2.72% at 0.2 mA/cm^2^ (Fig. 2e, Supplementary Fig. S2, Supplementary Table S2), which only falls below 1.5% at *V* > 10 V (i.e., *J* > 100 mA/cm^2^, the bias at which the radiance is maximised). Considering that the fraction of photons emitted above the 700 nm threshold is 43%, thus yielding a maximum “NIR EQE” of 1.17%, the efficiency we report here is among the highest values ever obtained for OLEDs based on a narrow-gap fluorescent emitter^26^.

Importantly, we note that although some degree of triplet harvesting (via TTA and/or rISC) occurs in the host-F8BT matrix^47–49^, as revealed by the transients recorded after application of rectangular voltage pulses (Fig. 2f, Supplementary Fig. S2), the relative weight of the delayed emission component is <10%, and it therefore does not compromise the OLED bandwidth and the overall VLC performance. Approximately 98% of the EL signal decays in less than 1 μs, albeit at an ~1000× slower rate than the PL dynamics. Such an apparent discrepancy, however, is due to the RC constant, which is the main limiting factor for high-frequency operation in OLEDs^9–14^. The effect of the parasitic RC is proved by the fact that we could measure some residual current (|*J*(*t*)|, black dotted lines in Fig. 2f and Supplementary Fig. S2f) flowing across the circuit up to ~1.5 μs after the pulse end.

### OLEDs bandwidth characterisation

**Fig. 3:**
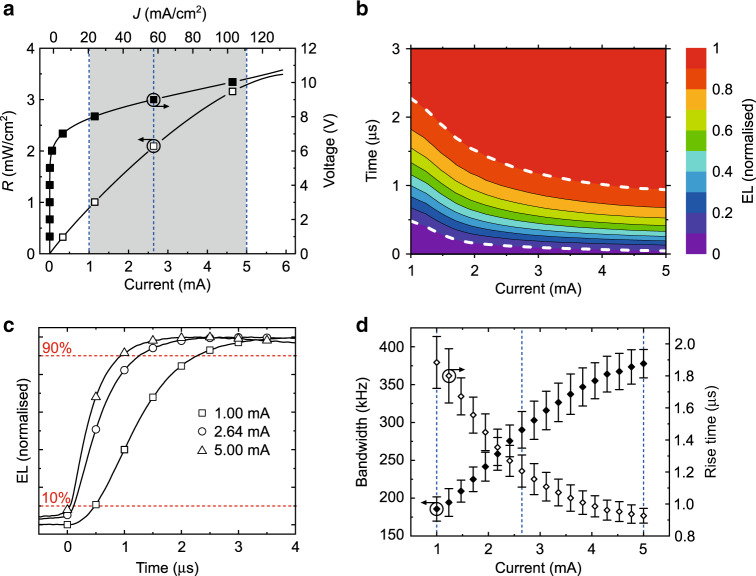
Rise time and bandwidth of F8BT:eDPP OLEDs with 0.2 wt% eDPP loading. **a** Typical radiance–current–voltage (*RIV*) curves of a 0.2% eDPP OLED. The grey shading highlights the DC operating bias range over which the OLED rise time was measured. **b** Contour plot showing the evolution of the OLED normalised (and spectrally integrated) EL intensity vs. operating current (on the horizontal axis) and vs. time from the square wave rising edge (on the vertical axis). The white dashed contour lines correspond to 10% and 90% of the modulated EL intensity (normalised to its maximum). **c** EL intensity vs. time plots measured at 1, 2.64 and 5 mA (4.5 mm^2^ device area) used for the determination of the so-called “rise time” corresponding to each rising edge of the square wave (applied at time 0). **d** Bandwidth vs. operating voltage calculated from the measured rise time values. The vertical dashed blue lines in **a** and **d** indicate the three different current values (1, 2.64 and 5 mA) at which the devices were operated in the real-time VLC experiment

Prior to integration of the F8BT:eDPP OLEDs into the VLC experimental setup illustrated in Fig. 1a, which is discussed in more detail in the Methods section, we characterised the dependence of their modulation bandwidth (BW) on the operating DC bias (Fig. 3, Supplementary Fig. S3).

As shown in the radiance–current–voltage (*RIV*) plot in Fig. 3a, for the 0.2 wt% blend, we selected a DC bias range from 1 to 5 mA (22–111 mA/cm^2^), and a 2 V peak-to-peak (Vpp) square wave was chosen to drive the OLEDs in a pseudo-linear operating region of the radiance–current (density) response at all times. The modulated EL intensity at the square wave rising edge as a function of time and DC bias is illustrated in Fig. 3b. Here, the contour lines corresponding to 10% and 90% of the normalised amplitude are highlighted to show the decrease in the characteristic rise time (*t*_r_) with increasing DC bias, from ~1.8 μs at 1 mA to ~0.9 μs at 5 mA. Such a reduction in the OLED response time is due to the decrease in the dynamic resistance of the diode and therefore in the RC constant with increasing current. The same trend can also be observed in Fig. 3c, which shows three EL intensity curves measured at the minimum, approximate centre and maximum bias points of the operating region, and in Fig. 3d, which shows *t*_r_ and BW (calculated as ~ 0.35*t*_r_^−1^) as a function of the bias.

Based on the observation of the maximum *BW* values measured among the different blends (Fig. 3d, Supplementary Fig. S3), we obtained the best performance for the 0.2 wt% OLEDs, which exhibit a maximum BW of 390 kHz at a 5 mA DC bias. Such a value is in line with the BW values previously reported by us^10–12,14^ and in principle could be exceeded by increasing the driving current beyond the quasi-linear operating range. However, it is noteworthy that the tendency of increasing BW with increasing bias seems to tail off at ~400 kHz for currents higher than 5 mA (i.e., 111 mA/cm^2^), above which the efficiency roll-off of the OLED EQE becomes significant (Fig. 2e).

For this reason, in the real-time VLC experiments, we tested the data transmission rate of each OLED at three different bias values, corresponding to the minimum, intermediate and maximum BW reported in Fig. 3 and Supplementary Fig. S3.

### OLEDs in real-time VLC

**Fig. 4:**
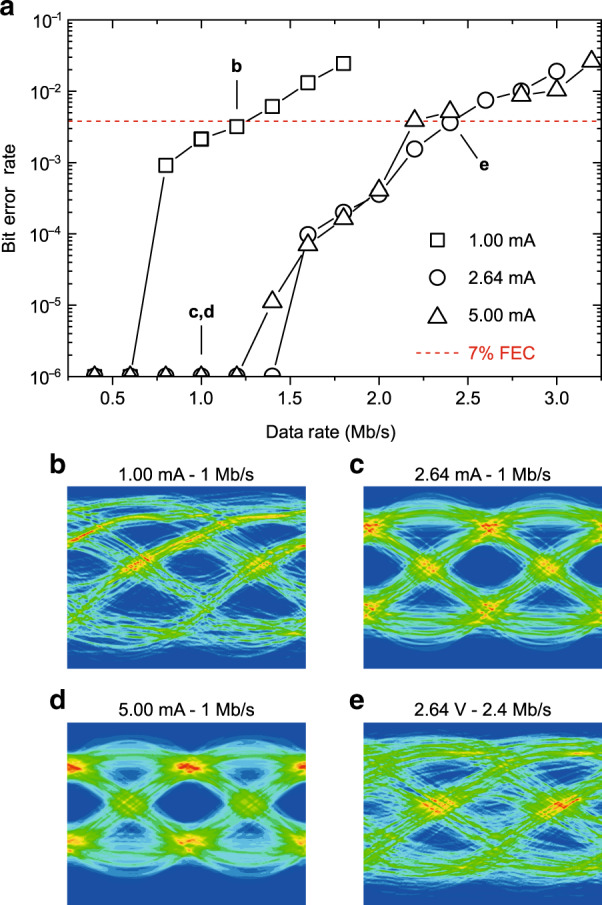
Bit error rate (BER) performance of F8BT:eDPP OLEDs with 0.2 wt% eDPP loading. **a** BER vs. data rate at 1, 2.64 and 5 mA operating biases. The BER limit for forward error correction (FEC) with a 7% data rate overhead is given as 3.8 × 10^−3^ and is indicated by the red dashed line. **b**–**d** Eye diagrams measured at a 1 Mb/s data rate with 1 (**b**), 2.64 (**c**) and 5 mA (**d**) DC biases. **e** Eye diagram measured at 2.4 Mb/s with a 2.64 mA DC bias. As highlighted by the arrows in (**a**), the diagrams in **b** and **e** correspond to a BER close to the 7% FEC threshold

Figure 4 shows the measured real-time bit error rate (BER) for the 0.2 wt% F8BT:eDPP OLEDs measured as a function of data rate.

Referring to Fig. 1a and the Methods section, where the full system description is given, one field programmable gate arrays (FPGAs) hosted in National Instruments reconfigurable Universal Software Radio Peripherals (USRPs) were used to generate a series of pulses emulating data traffic in the on-off keying transmission mode. Next, the data were up-sampled and pulse shaped using a 15% excess energy square-root raised cosine before conversion to an analogue signal. Then, the signals were biased via a current mirror that set the bias current to the mentioned values and the signal swing to 2 Vpp before transmission over the free-space VLC link. The receiver consisted of a ThorLabs PDA36A-2 silicon detector, followed by digitisation, synchronisation and matched filtering via a time-reversed copy of the transmit filter for protection from inter-symbol interference (ISI) and maximisation of the signal-to-noise ratio before detection.

The system under test was capable of supporting transmission under the 7% forward error correction limit (i.e., BER = 3.8 × 10^−3^, red dashed line) up to 2.4 and 2.2 Mb/s for the 2.64 and 5 mA systems, respectively, corresponding to actual rates of ~2.2 and 2 Mb/s, respectively, after overhead removal. It may be expected that increasing the bias would result in higher transmission speeds due to the increased bandwidth availability (as shown in Fig. 3). However, with increasing bias, the non-linearity also increases due to the EQE roll-off with current, as discussed in the previous section, which results in a small BER penalty and reduced data rate availability. When the system is under-biased (at 1 mA, i.e., just above turn-on), a reduced gross (net) rate of ~1.25 (~1.1) Mb/s is available since the on–off transmission is significantly slower due to the substantial bandwidth reduction (Fig. 3d).

This is the first demonstration of such a system, and the rates reported here represent a world record for real-time data transmission using OLEDs.

## Discussion

We have demonstrated red-to-NIR (650–800 nm) OLEDs with efficiencies exceeding 2.7%, a maximum radiance of 3.5 mW/cm^2^ and luminance up to 260 cd/m^2^, having as the active material a structurally unique π-expanded diketopyrrolopyrrole (eDPP) embedded in a polyfluorene-based host matrix (F8BT). The efficiency we report here is remarkable considering the “heavy-metal-free” polymeric nature of the active layer, the reduced efficiency roll-off and the relatively low energy gap of the eDPP emitter.

We used such devices to achieve a major breakthrough by incorporating them into a real-time VLC setup, showing error-free operation at data rates exceeding 2 Mb/s, which are unprecedented for an online unequalised VLC link based on organic LEDs and high enough to support, for example, an indoor point-to-point link (e.g., with a view to IoT applications). The possibility of achieving such data rates without computationally complex and power-demanding equalisers, together with the absence of toxic heavy metals in the active layer of the OLEDs, is promising for the integration of portable, wearable or implantable organic biosensors into visible/NIR light communication links.

## Materials and methods

### Synthesis

Poly(9,9-dioctylfluorene-*alt*-benzothiadiazole) (F8BT) was purchased from American Dye Source (ADS133YE). The eDPP dye was synthesised in seven steps starting from fluorene, following our previously reported procedure^36^. Cyclic voltammetry (CV) of eDPP (1 mg/mL) is reported in Section S4 of the Supplementary information file. Cyclic voltammograms were measured at 20 °C under an argon atmosphere in a deoxygenated 0.1 M solution of tetrabutylammonium perchlorate in anhydrous dichloromethane. A glassy carbon working electrode, a Ag/AgCl reference electrode, and auxiliary platinum foil were used, while the scan rate was set at *v* = 100 mV s^−1^. The highest occupied molecular orbital (HOMO = −5.2 eV) level of eDPP was extracted from the first ionisation potential, measured via CV. Due to the weak reduction signal, the lowest unoccupied molecular orbital level (LUMO = −3.4 eV) of eDPP was calculated from knowledge of the HOMO and the optical energy gap, extrapolated from the absorption edge of eDPP.

### PL characterisation

For optical characterisation, a 10^−6^ M solution of eDPP in chloroform was prepared in a fluorescence quartz cuvette with a 10 mm optical path length. F8BT:eDPP thin films were deposited via spin-coating at 1500 rpm onto fused silica substrates from 10 mg mL^−1^ toluene solutions in an N_2_-filled glove box to obtain a thickness of ~125 nm (measured with a Dektak profilometer). The absorption spectra were measured by using an Agilent 8453 UV-Vis spectrometer. The PL spectra were collected with an Andor Shamrock SR-163 spectrograph (with the Czerny-Turner optical layout, a 300 lines/mm 500 nm blazed grating, a 163 mm focal length, and an f/3.6 numerical aperture) coupled to a silicon-based Andor Newton electron multiplying charge-coupled device with a maximum resolution of 0.77 nm. All PL (and EL) spectra were corrected for the spectral responsivity of the instrument. The PL efficiency was measured with an integrating sphere setup, following the protocol reported in ref. ^53^. Time-resolved fluorescence measurements were carried out with a time-correlated single photon counting (TCSPC) spectrometer (Edinburgh instruments, LifeSpec II). For both steady state and transient fluorescence experiments, the eDPP solution was excited using a 375 nm ps pulsed LD (Edinburgh Instruments EPL 375, 20 MHz repetition rate). For thin-film characterisation, a 445 nm pulsed LD (EPL 445) was used to excite mainly the F8BT host matrix, with only marginal absorption by eDPP.

### OLED fabrication

The ITO/PEDOT:PSS/F8BT:eDPP/Ca/Al OLEDs reported here were fabricated on commercial (OSSILA Ltd) ITO substrates (anode), which were treated in an oxygen plasma chamber for 10 min^54^. To facilitate hole injection, the ITO substrates were covered with a 40 nm layer of PEDOT:PSS, which was spin-cast from a 2.8 wt% water dispersion (Sigma-Aldrich) and annealed at 150 °C for 10 min in the glove box. After annealing, the active F8BT:eDPP blends (~125 nm) were spin coated on top of the PEDOT:PSS layer from the same solutions used for the PL experiments. Following this, 30 nm of calcium and 200 nm of aluminium were thermally evaporated on top of the emitting polymer layer to serve as a cathode. Finally, the OLEDs were encapsulated in the glove box by depositing a UV-curable epoxy glue (OSSILA Ltd) layer covered by a protective glass slide. Each device consisted of six independent pixels with an active area of 4.5 mm^2^ per device as defined by the overlap between the ITO (anode) and Ca/Al (cathode).

### EL characterisation

OLEDs were characterised using a Keithley 2400 source meter for both the voltage supply and the current measurement. The optical output of the OLEDs was measured using a calibrated silicon photodiode, while the EL spectra were collected with the same spectrometer used for the PL experiments.

### Time-resolved EL characterisation

Time-resolved EL measurements were carried out by biasing the OLEDs with an Agilent 8114A pulse generator. The pulse width was set at 1 μs, whereas the pulse repetition rate was varied between 49.67 and 208 kHz. Photons emitted from the devices were collected using an f/4, 300 mm, Acton Research Spectra Pro SP-2300i triple-turret monochromator with a Hamamatsu H7422-20 Peltier-cooled photosensor module as the detector. EL transients were measured by means of PicoQuant TimeHarp-100 and PicoQuant NanoHarp-250 electronic boards synchronously driven by the pulse generator. The transient current flowing across the circuit during the OLED pulsed operation was also monitored. This signal was obtained by measuring the transient voltage drop across a calibrated resistor (*R* = 47 Ω, with a GWINSTEK GDS-2204 oscilloscope) in series with the OLED.

### Bandwidth characterisation

The bandwidth was characterised based on the transmission of a 50 kHz square wave generated using a Tektronix AWG70002 arbitrary waveform generator, which supplied a 0.5 Vpp signal amplified by a Texas Instruments (TI) THS2302 by a factor of 4. The signal was biased with a DC current ranging from 1 to 5 mA and an AC voltage of 2 Vpp. The transmission of the square wave in free space was detected at a distance of 5 cm via a ThorLabs PDA36A-2, which had a bandwidth of 12 MHz and a 0-gain built-in transimpedance amplifier. Next, the signal was amplified by a factor of 40 using cascaded TI THS3202 amplifiers. The signals were then digitised by a Tektronix real-time oscilloscope (MSO70804C). Approximately 10 M samples were captured at a sampling rate of 62.5 MS/s via a computer interface and imported to MATLAB, where the rise times were measured.

### VLC setup and protocol

The VLC setup, referring to the simplified block diagram in Fig. 1a, consisted of two FPGAs embedded within NI USRPs. At the transmitter, an NI 2953R is used to generate a pseudo-random binary sequence (PRBS) of length 2^15^−1 before up-sampling via zero padding at a rate of *N*_ss_ = 4 samples per symbol. The symbols are then passed through a square-root raised cosine pulse shaping filter with 15% excess energy, which is implemented via a finite impulse response (FIR) filter that spans a length of *L*_s_ = 12 symbols to avoid the introduction of symbol distortion as described earlier^55^. Therefore, the total number of taps in the FIR filter (given as *L*_s_*N*_ss_ + 1) is 49. After pulse shaping, the signal is then converted for modulation via an Ettus Research LFTX digital-to-analogue converter (DAC), which has a 30 MHz BW and 12 vertical bits, before modulation of the OLED via a current mirror circuit designed to supply the previously mentioned bias current and voltage swing. The distance between the OLED and the photodetector was set to 5 cm, and the photodetector used was as described above and included the 40 times amplification (given the >2M pixels present in 1080p high-definition displays, we expect that the envisaged display technology application will provide sufficient data rate operation over a distance of several cm to metres). At the receiver, an NI USRP 2943 along with an Ettus Research LFRX analogue-to-digital converter with the same characteristics as the LFTX is used to digitise the signal before synchronisation via edge detection is performed to give best-effort signal alignment. A time-reversed matched square-root raised cosine FIR filter with specifications equivalent to those of the previous filter is used to maximise the signal-to-noise ratio as much as possible before down-sampling at the midpoint of the symbol and comparison with the transmitted bits in a symbol-by-symbol manner to measure the BER.

## Supplementary information

Supplementary information - Visible Light Communications with Efficient Far-Red/Near-infrared Polymer Light-emitting Diodes
