# Supplementary material for: Visible light communication with efficient far-red/near-infrared polymer light-emitting diodes
Source: Light Sci Appl. 2020 Apr 26;9:70. doi: 10.1038/s41377-020-0314-z (PMC7183573; doi:10.1038/s41377-020-0314-z)
Supplement: Supplementary file 1 — Supplementary information - Visible Light Communications with Efficient Far-Red/Near-infrared Polymer Light-emitting Diodes [file 41377_2020_314_MOESM1_ESM.pdf]

Supplementary information

Visible Light Communication with Efficient Far-Red/Near-Infrared Polymer Light-emitting Diodes

Alessandro Minotto[1], Paul A. Haigh[2,3], Łukasz G. Łukasiewicz[4], Eugenio Lunedei[5], Daniel T. Gryko[4*], Izzat Darwazeh[3*] and Franco Cacialli[1*]

[1]Department of Physics and Astronomy and London Centre for Nanotechnology, University College London, London, WC1E 6BT, UK

[2]School of Engineering, Newcastle University, Newcastle-upon-Tyne, NE1 7RU, UK

[3]Communications and Information Systems, University College London, London, WC1E 6BT, UK

[4]Institute of Organic Chemistry, Polish Academy of Sciences, 01-224 Warsaw, Poland

[5]ISMN-CNR, Institute for the Study of Nanostructured Materials, 40129 Bologna, Italy

*e-mail: dtgryko@icho.edu.pl, i.darwazeh@ucl.ac.uk, f.cacialli@ucl.ac.uk

Table of contents

1. Optical characterisation of F8BT:eDPP blends 2

2. F8BT:eDPP OLEDs 7

3. Bandwidth characterisation 9

4. Cyclic voltammetry data 10

References 11

1. Optical characterisation of F8BT:eDPP blends

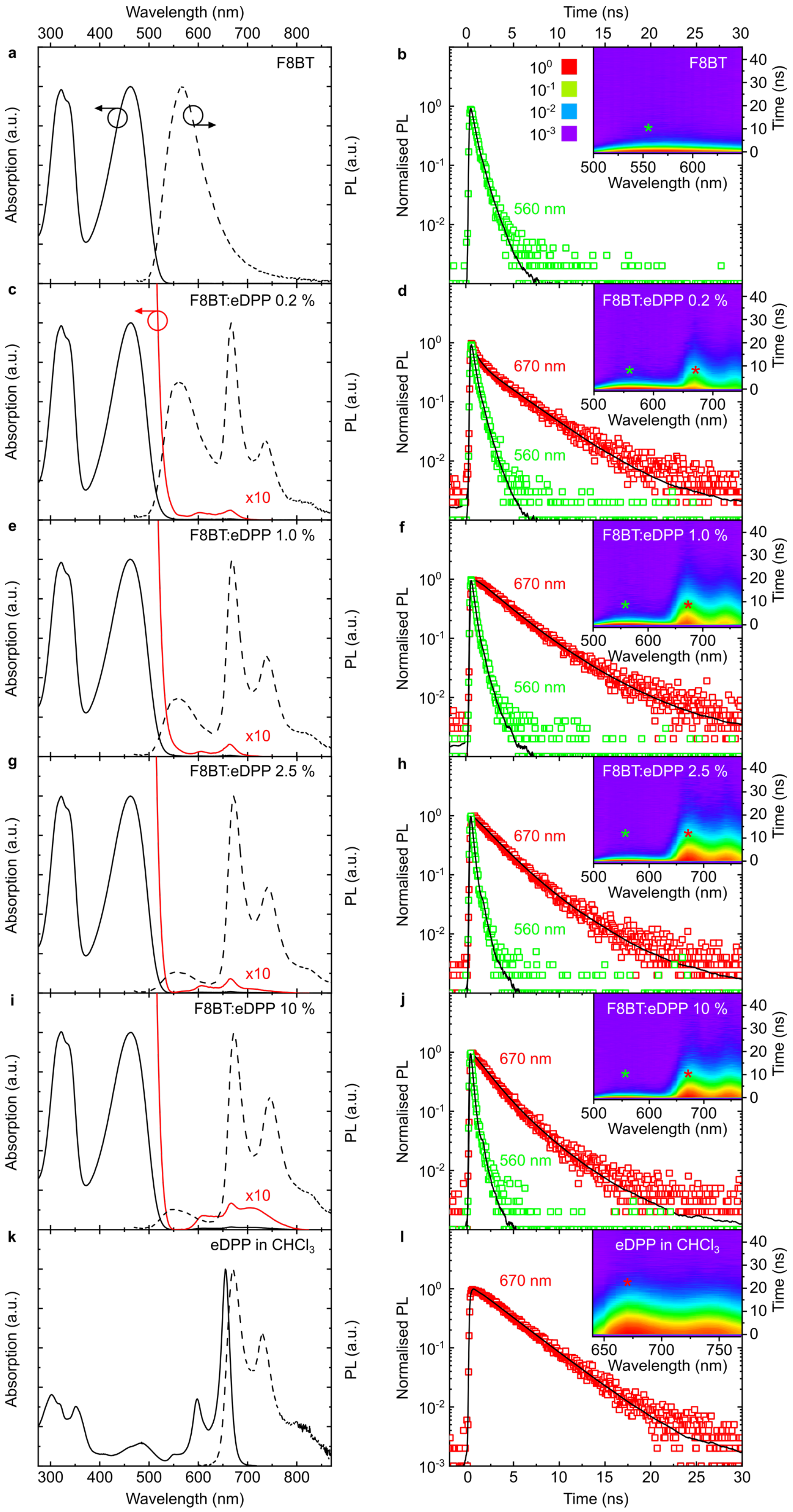

Fig. S1. Absorption spectra, PL spectra and transient PL of F8BT, F8BT:eDPP blends thin films with 0.2, 1, 2.5 and 10 wt% of eDPP loading and eDPP in chloroform (~ 10^{-6} M).

In Fig. S1 we report the normalised absorption and photoluminescence (PL) spectra of the F8BT host matrix, F8BT:eDPP blends thin films (Fig. S1a,c,e,g,i) at different eDPP loadings and of eDPP in chloroform solution (Fig. S1k), together with the corresponding normalised time-resolved PL and spectra (Fig. S1b,d,f,h,j,l).

To highlight the absorption from the eDPP dopant in the blends, we multiplied the corresponding spectra by a factor of ten (red lines in Fig S1c,e,g,i). For all blends the eDPP absorption peaks at 665 nm, which is ~ 10 nm red-shifted compared to the absorption maximum as observed in $CHCl_3$ diluted (~10^{-6} M) solution, with a broader band in the 700 – 800 nm range (peaking at ~ 725 nm) not observed in solution. We ascribe this to the absorption from eDPP aggregates, as its relative intensity rises with increasing eDPP concentration in the F8BT matrix.

Moving to the PL spectra (black dashed lines), the emission from the F8BT host (peaking at 560 nm) is progressively quenched at increasing dopant concentration, due to the concomitant enhancement of the resonant energy transfer (ET) rate to the eDPP red/NIR emitters. The fluorescence from eDPP in the blends is peaked at 670 nm, as for the solution, with secondary vibronic components at 740 and 800 nm, respectively. The most evident variation observed across the F8BT:eDPP blend series is the relative increase with eDPP concentration of the 0-1 vibronic transition at 740 nm, as a result of the oscillator strength redistribution induced by the intermolecular interactions between chromophores. However, such a change in the spectral profile is in part accounted for by self-absorption originating from aggregates in the films with higher eDPP loading, exhibiting absorption at λ > 700 nm.

In Table S1 we summarise the PL efficiency (Φ) and the fraction of photons emitted from the eDPP dopant (f^{eDPP}) for thin films (on Spectrosil, measured at room

temperature) of all blends and neat materials, together with the PL lifetimes (where we use the notation $\tau_i^{xxx\ nm}$ to indicate the *i-th* lifetime measured at a wavelength of xxx nm) and the energy transfer efficiency (η^{ET}) extracted from the transient PL in Fig. S1, as well as the theoretical maximum PL efficiency Φ^{th}

$$\phi^{th} = \phi^{eDPP}\eta^{ET} + \phi^{F8BT}(1-\eta^{ET}) = 0.93\eta^{ET} + 0.22(1-\eta^{ET})$$

where ϕ^{eDPP} is the PL efficiency of eDPP in dilute (~10^{-6} M) solution, and ϕ^{F8BT} is the PL efficiency of an F8BT neat film on spectrosil.

Table S1. Summary of the main PL properties (All data are for thin films on Spectrosil, at room temperature, except for the last line referring to a 10^{-6} M $CHCl_3$ solution).

	Φ [%]	f^{eDPP} [%][a)]	$\tau_1^{560\ nm}$ [ns][b)]	$\tau_2^{560\ nm}$ [ns][b)]	$\tau_1^{670\ nm}$ [ns][b)]	$\tau_2^{670\ nm}$ [ns][b)]	η^{ET} [%][c)]	Φ^{th} [%][d)]
F8BT	22 ± 1	-	0.39 (60%)	0.97 (40%)	0.50 (76%)	1.81 (24%)	-	
0.2% eDPP	31 ± 2	52	0.35 (73%)	0.97 (27%)	**3.86 (83%)**	0.51 (17%)	17	34
1.0% eDPP	37 ± 2	78	0.31 (81%)	0.97 (19%)	**3.16 (77%)**	6.31 (23%)	30	43
2.5% eDPP	45 ± 3	93	0.23 (87%)	0.97 (13%)	**2.55 (83%)**	6.31 (17%)	48	56
10% eDPP	33 ± 2	94	0.19 (88%)	0.97 (12%)	**2.09 (84%)**	6.31 (16%)	54	60
eDPP in $CHCl_3$	93 ± 5	-	-		**3.68**		**-**	

[a)] Fraction of photons emitted at λ > 635 nm. b) Percentage weights are reported between parentheses. $\tau_i^{xxx\ nm}$ indicates the *i-th* lifetime measured at a wavelength of xxx nm. c) Efficiency of resonant energy transfer (ET) from the F8BT host to the eDPP dopant.

We extracted the ET efficiency (η^{ET}) reported in Table S1 from the quenching of the F8BT emission when doped with eDPP, following the relation[1] $\eta^{ET} = 1 - \tau^*/\tau$, with τ corresponding to the PL average lifetime of F8BT as neat film, and τ^* as the average PL lifetime of the F8BT emission from the blends with eDPP (here the average is a weighted average of the fitted decay lifetimes with the weights as extracted from the fits for both τ and τ^*). These lifetimes were obtained by fitting the PL decay via iterative reconvolution of a bi-exponential equation with the instrument response function. Fits are shown in Fig. S1 as black lines.

We measured the highest Φ (45%) from the 2.5 wt% blend, thanks to the efficient ET from the green-emitting F8BT host to the efficient eDPP red/NIR emitters (note the outstanding Φ =93% in chloroform solutions), despite some aggregation quenching being present at this dopant concentration, as discussed in the main manuscript. Efficient ET affords high red/NIR colour purity, with a residual green emission from F8BT representing only 7% of the total PL integral (Table S1). By increasing the eDPP concentration aggregation quenching also increases, leading to an overall lower Φ. By reducing the eDPP concentration, instead, both the PL efficiency (Φ) and the PL colour purity decrease, due to less efficient ET and higher residual emission from F8BT, whose intrinsic Φ is only 22% (vs. 93% for eDPP).

Looking at the eDPP time-resolved PL in chloroform solution, we observe a mono-exponential decay with 3.68 ns characteristic lifetime ($\tau_1^{670\ nm}$ in Table S1). We extracted a similar time constant ($\tau_1^{670\ nm}$ = 3.86 ns) from the 0.2 wt% blend, with the ~ 0.2 ns increase most likely due to the reduction of the radiative rate induced by the interaction with the conjugated polymeric matrix. Differently from the eDPP solution, however, an additional faster component ($\tau_2^{670\ nm}$ = 0.51 ns, weight = 17%) arising from the residual F8BT emission could be detected from the PL decay of the 0.2 wt% blend at 670 nm.

The contribution from F8BT (residual) emission at 670 nm becomes negligible, however, already at 1 wt% eDPP concentration, due to a higher energy transfer efficiency η^{ET}. This is associated with an overall increase of the PL lifetime with respect to the 0.2 wt% blend. Nevertheless, the PL decays of the 1, 2.5 and 10 wt% films still exhibit a bi-exponential trend, with characteristic lifetimes and percentage weights reported in Table S1. The main difference compared to the PL decay from the eDPP dye in solution and in the 0.2 wt% blend is the occurrence of a

6.31 ns long component ($\tau_2^{670\ nm}$), extracted from the global fit of the PL decays measured from the 1, 2.5 and 10 wt% blends thin films. This component, with percentage weight < 25%, can be ascribed to the weakly emissive eDPP aggregates absorbing at λ > 700 nm.

Yet, despite the presence of these intermolecular species, the almost invariant spectral shape across the different blends and the eDPP solution suggests that most of the emission in the red/NIR region originates from isolated (unaggregated) eDPP fluorophores. Indeed, from a global bi-exponential fit of all decays collected in the 670 nm – 770 nm range, the contribution from τ_1 is always > 75%. However, we note also that at increasing eDPP concentration the $\tau_1^{670\ nm}$ value decreases from 3.86 to 2.09 ns (for the 0.2 and 10 wt% blend, respectively). We attribute such a decrease to the presence of ET from the isolated eDPP to the poorly emissive intermolecular species, whose absorption at λ > 700 nm spectrally overlaps with the emission of the unaggregated dye.

2. F8BT:eDPP OLEDs

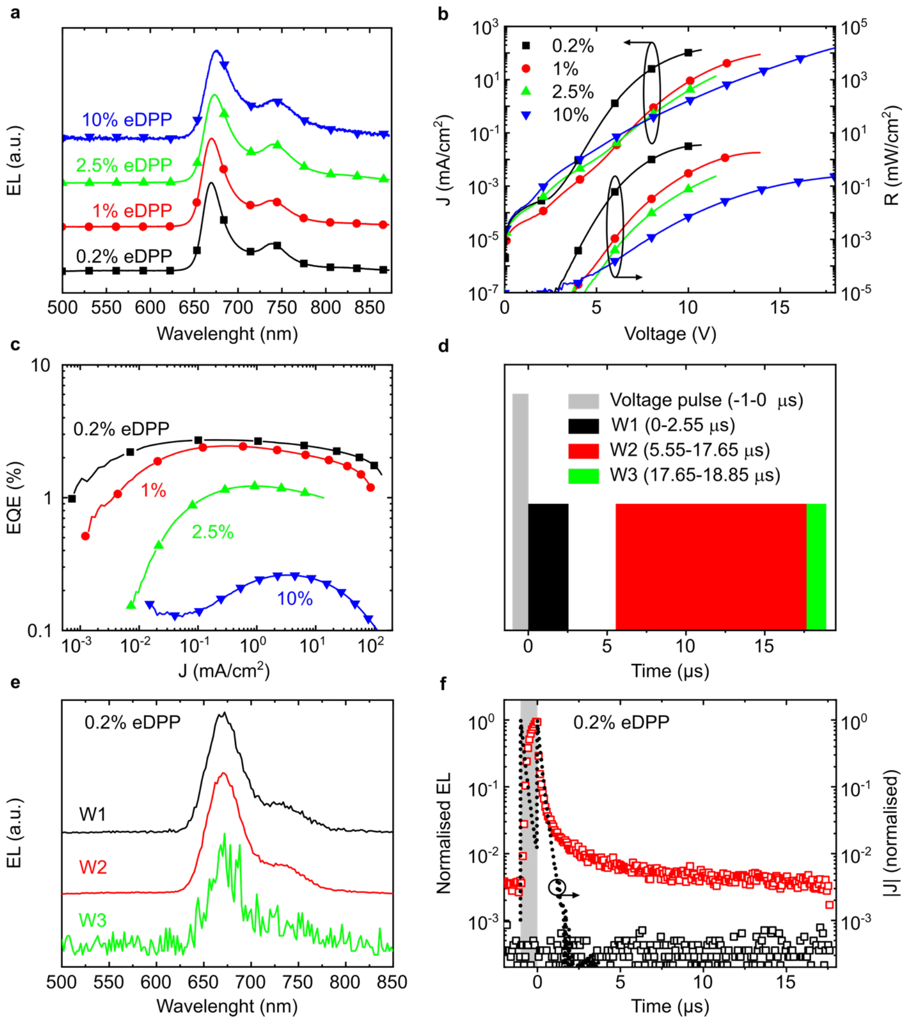

Fig. S2. **a,b,c**, EL spectra (**a**), JVR characteristics (**b**) and EQE versus J plot (**c**). The EL spectra in **a** were measured at the bias at which the EQE is maximised. **d**, Scheme illustrating the time windows during which the time-resolved EL spectra have been measured. **e**, Normalised spectra collected by integrating the EL signal from a 0.2 wt% F8BT:eDPP OLED within the W1, W2 and W3 time windows. Operation of the OLEDs with rectangular voltage pulses revealed that EL could be detected at several μs after the voltage pulse end with no variation of the spectral shape over time, as expected in the presence of TTA and/or rISC. **f**, Normalised transient EL at 670 nm and background signal (red and black squares, respectively, both normalised for the EL maximum) measured by exciting the 0.2 wt% OLEDs with 4.8 V rectangular voltage pulses (50 kHz repetition rate, 1 μs pulse width). The dotted line in **f** represents the absolute value of the normalised transient current (|J(t)|) flowing across the circuit during the OLED pulsed operation. We note here that since no current could be measured across the circuit already at ~ 1.5 μs after the pulse end (dotted line in **f** and Fig. 2f of the main manuscript) we can rule out that delayed emission could originate from the residual current after the switch-off of the driving voltage.

Table S2. Summary of DC performance of the F8BT:eDPP OLEDs.

	V_{ON} [V]	EQE_{MAX} [%]	Radiance mW cm^{-2}
0.2% eDPP	3.3 ± 0.6	2.57 ± 0.12	3.14 ± 0.49
1.0% eDPP	3.5 ± 0.5	2.02 ± 0.39	1.45 ± 0.32
2.5% eDPP	4.1 ± 0.3	1.17 ± 0.04	0.67 ± 0.24
10% eDPP	3.5 ± 0.5	0.24 ± 0.03	0.34 ± 0.09

3. Bandwidth characterisation

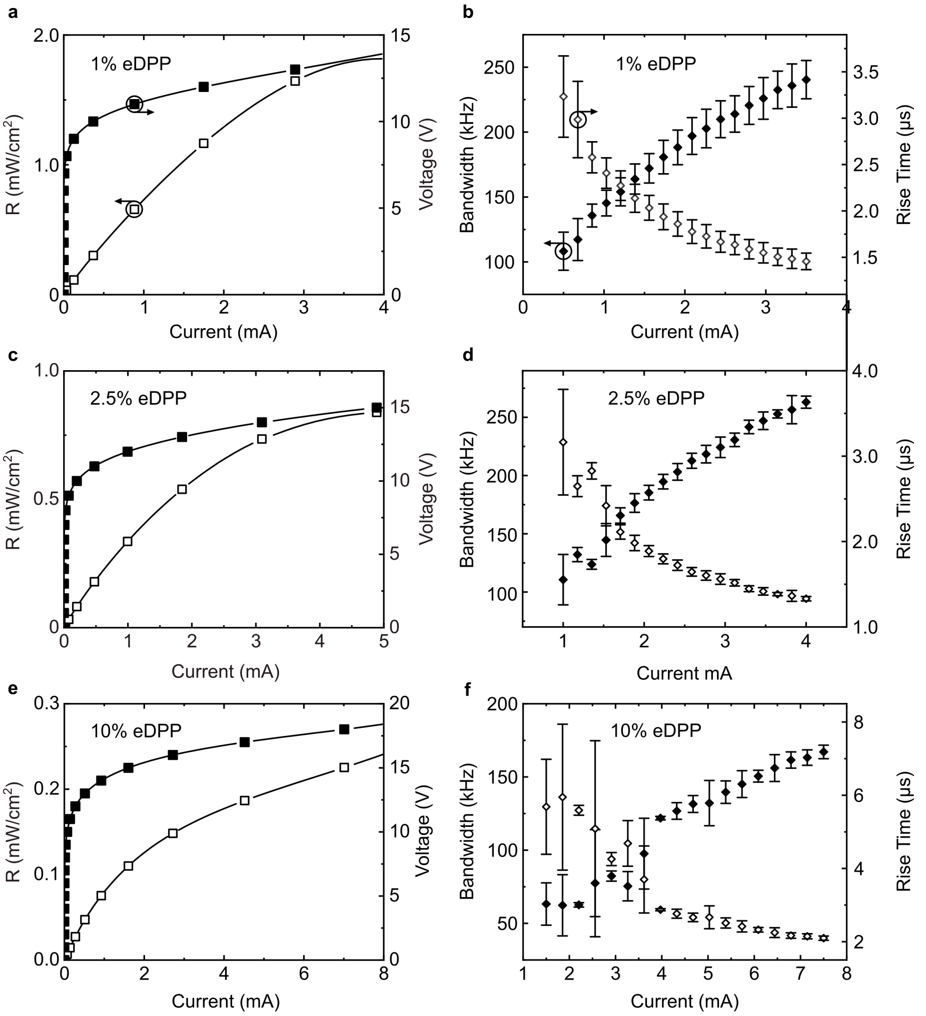

Figure S3. ***a,c,e***, *Typical Radiance-Current-Voltage (RIV) curves of a 1%, 2.5% and 10% eDPP OLED.* ***b,d,e***, *Bandwidth and rise time versus operating bias.*

4. Cyclic voltammetry data

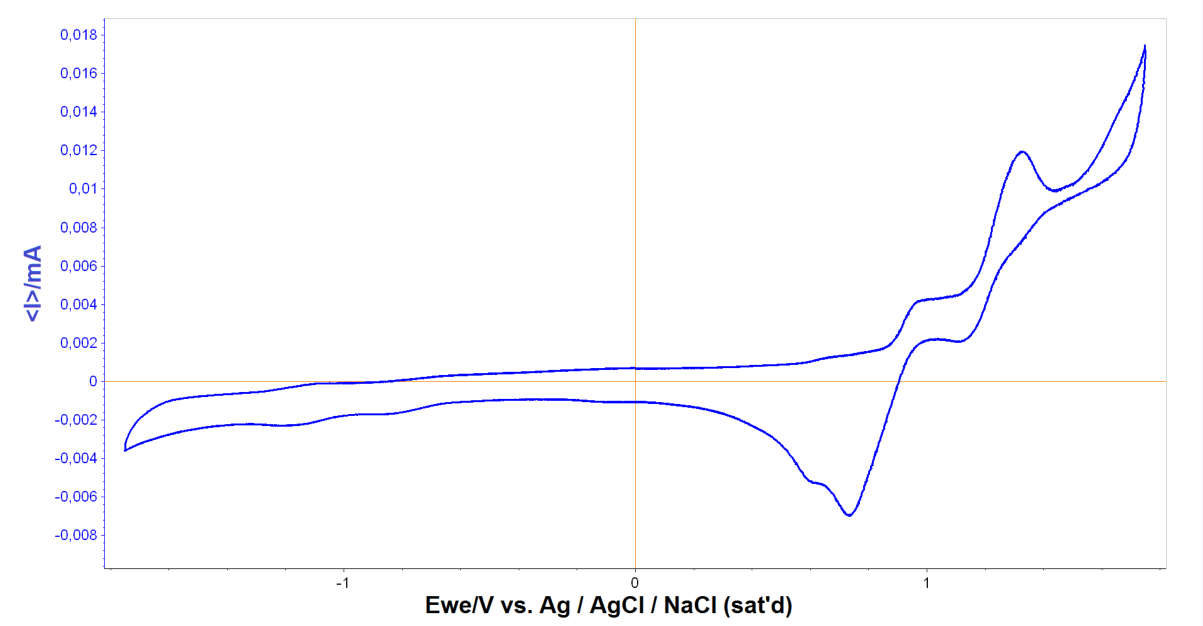

Figure S4. *Cyclic voltammetry (CV) of eDPP at room temperature.*

Cyclic voltammograms of eDPP (1 mg mL^{-1}) were measured at 20°C, under an argon atmosphere, in deoxygenated 0.1 M solution of tetrabutylammonium perchlorate in anhydrous dichloromethane. A glassy carbon working electrode, a Ag/AgCl reference electrode and auxiliary platinum foil were used, while the scan rate was set at v = 100 mV s^{-1}. All values of E [V] are summarised in the table below with respect to Fc^+/Fc redox potential.

$E^1_{ox}{}^{pa}$ [V]	$E^1_{ox}{}^{pc}$ [V]	$E^2_{ox}{}^{pa}$ [V]	$E^1_{ox}{}^{pc}$ [V]	$E^1_{ox}{}^{onset}$ [V]	$E^2_{ox}{}^{onset}$ [V]	IP^1 [eV]	IP^2 [eV]
0.97	0.74	1.33	1.13	0.87	1.17	-5.21	-5.51

References

1. Lakowicz, J. R. *Principles of Fluorescence Spectroscopy*. (Springer US, 2006).
